# Through the Ivory Gate

**Published:** 1890-03

**Authors:** 


**Through the Ivory Gate**. Studies in Psychology and History, by W. W. IRELAND, M. D., Edin., formerly of H. M. Indian Army; corresponding member of the Psychiatric Society of St. Petersburg, and of the New York Medico-Legal Society; author of "The Blot on the Brain." 8vo, pp. New York: G. P. Putnam's Sons. Edinburgh: Bell & Bradfute. 1889.

In his short preface, the author says:

This book is written in prosecution of the views stated in "The Blot on the Brain." The historical and psychological studies may be considered as a continuation of the papers in that book on Mohammed, Joan of Arc, Mohammed Toghlak, and others. All the characters described in the present work, in my opinion, suffered from mental derangement. They were led away by delusions of uncontrollable passions from the right comprehension of things, or the right line of conduct. In figurative language, they were visited by spectres which passed through the Ivory Gate.

Quoting from Pope's translation of the Odyssey, he says:

Immured within the silent bower of sleep,
Two portals firm the various phantoms keep;
Of ivory one; whence flit to mock the brain,
Of winged lies a light fantastic train:
The gate opposed pellucid valves adorn,
And columns fair incased with polished horn,
Where images of truth for passage wait,
With visions manifest of future fate.

Thus the aim of the work is tersely and even practically stated at the very threshold, and the reader, wholly relieved from doubt as to its intellectual purpose, can follow him with the full enjoyment a well-written book of absorbing interest may yield him. The only thing left for the critic is the inquiry: Have the facts and premises on which his conclusions were based been stated with fairness and without undue prejudice, and do his deductions follow with sufficient probability in the light of the physiological science of the day? To the first clause of this question the unprejudiced reader must give his unqualified assent; the answer to the latter must necessarily depend in a great degree upon the quality of his mind, his preconceived ideas, and his will or ability to divest himself of them in the interest of science.

The first, and most important study, is the life, character, and mental state of Swedenborg. He shows him to have been, at least in the earlier years of his life, all that his intelligent admirers claim, but that he inherited a neurotic temperament which late in life developed into a real mental derangement. He says:

We have seen that after a period of great nervous excitement at the Hague,

Swedenborg had in London, in 1744, an acute attack of insanity, (*paranoia acuta hallucinatoria ?*) This calmed down in a few months, and gradually the will and intellect resumed their power, though not to struggle against the delusions that had taken hold on his mind, but finding meaning in them to systematise them and to propagate them. * * * He remained the rest of his life in a state of delusional insanity, or paranoia.

From the grouping of apparently well authenticated facts in the history of this remarkable man, it is difficult, if not impossible, for the student of nervous maladies to arrive at any other explanation of his theological writings than that they were the offsprings of a highly cultivated mind, naturally independent in thought, self-sufficient and fearless in expression, and with a tendency to systematisation mingled with vagaries proceeding from a "heat-oppressed brain." Swedenborg's spirits probably never told him a valuable truth that was not preëxisting in his own mind the result of previous sane and philosophical reflections. All the rest of his strange spiritual experience can easily be attributed to delusions.

The concluding remarks of the author are pertinent, as they serve to show the fair animus of his article. He says:

It is sad to think that it should have been the lot of so earnest a seeker after truth to wander so many years in the mazes of delusion; but the records of mental derangement contain some of the saddest things in fate. Swedenborg's moral and theological writings contain much that is noble and true, though marred by whimsical notions and erroneous statements. Nevertheless, many of the sayings committed to writing will find acceptance with thoughtful men, bearing their own evidence in their fitness to other things in the plan of the world.

William Blake is the next of the author's subjects, and though different in many respects from the Swedish Seer, his acts and notions are referred to the same category of mental diseases and as appears with justice.

A long and exhaustive study of the mental state of Giteau follows.

Probably no calm and unbiased specialist in nervous disease, with all the facts before him of the mental history of Giteau before the murder, his conduct afterwards during the protracted trial, and his extraordinary behavior at the execution, would be willing to pronounce him a sane man. At the same time, a candid review of the subject must lead to the conclusion that there was sufficient "method in his madness" to make him responsible for his act in the eye of the law. The author leans in this direction, though in some of his conclusions he differs from both Hammond and Gray, believing that, though under an abnormal condition of mind (paranoia?), the *intensity* of his delusion was not so strong as to have compelled his wicked act. He says:

Men like Giteau, at once wicked and wrong-headed, are not so very rare, and they need an example. * * * * Like many who come into the grasp of the law, he was the victim of hereditary tendencies and a bad education. A well-directed and watchful training would likely have turned the bias in a better direction. For the dismal result Giteau himself was not without blame. He yielded to temptation till he lost the reins of his own passions and caprices.

The whole of this interesting subject is ably handled, and will repay careful perusal by the general reader, as well as by the medical student.

Thirty-six pages are next devoted to the case of Riel, the Canadian rebel; it is an exceedingly interesting study. His was a very checkered career. He tries to prevent the absorption of Manitoba into the Dominion by force; is afterwards sent to Parliament, next into an insane asylum; has religious delusions, claims inspiration, heads a rebellion, fights a battle, is made prisoner, tried and executed. The plea of insanity was urged at his trial, and with apparent reason, but without avail, though many, our author included, hold that the ends of justice might have been equally served with a milder punishment.

Among recent trans-Atlantic lunatics are selected for study Louis II. of Bavaria; the Italian Jesuit Gabriel Malgrida; Theodore, King of Abyssinea, and others, all of which exhibit to a greater or less degree their aberration from the normal mental condition of Man.

In concluding this notice, the idea is forced upon the mind that the boundary line between our "IVORY GATE" and that with "valves pellucid," is somewhat indistinct. May not the blot on the brain be more common than we think?

